# A Prospective Study on Health-Related Quality of Life and Patient-Reported Outcomes in Adult Brain Tumor Patients Treated with Pencil Beam Scanning Proton Therapy

**DOI:** 10.3390/cancers13194892

**Published:** 2021-09-29

**Authors:** Stephanie G. C. Kroeze, Paul-Henry Mackeprang, Claudio De Angelis, Alessia Pica, Barbara Bachtiary, Ulrike L. Kliebsch, Damien C. Weber

**Affiliations:** 1 Center for Proton Therapy, Paul Scherrer Institute, Forschungstrasse 111, 5232 Villigen, Switzerland; stephanie.kroeze@usz.ch (S.G.C.K.); Paul-Henry.Mackeprang@insel.ch (P.-H.M.); claudio.deangelis@psi.ch (C.D.A.); Alessia.pica@psi.ch (A.P.); barbara.bachtiary@usz.ch (B.B.); ulrike.kliebsch@psi.ch (U.L.K.); 2 Department of Radiation Oncology, University Hospital of Zürich, 8091 Zürich, Switzerland; 3 Department of Radiation Oncology, Inselspital, Bern University Hospital, University of Bern, 3012 Bern, Switzerland

**Keywords:** brain tumors, HRQOL, patient-related outcomes, EORTC-QLQ-C30, EORTC-QLQ-BN20, proton therapy, pencil beam scanning, toxicity

## Abstract

**Simple Summary:**

Patients with complex brain tumors are regularly treated with proton therapy (PT) to obtain an optimal curative treatment with limited toxicity. Value-based oncological medicine is increasingly important, particularly when long-term survival is to be expected. The aim of our prospective study was to evaluate health-related quality of life (HRQOL) and patient reported outcomes (PROs) in patients treated with PT for brain tumors. PT only temporarily influences PROs and QoL directly following PT and lower QoL and PROs were mostly reported by patients who were already in a worse condition with symptoms related to the tumor and previous treatment, before the start of PT. This study assists in improving patient support in patients with brain tumors receiving PT.

**Abstract:**

Proton therapy (PT) is delivered to complex brain tumors to obtain an optimal curative treatment with limited toxicity. Value-based oncological medicine is increasingly important, particularly when long-term survival is to be expected. This study aims to evaluate health-related quality of life (HRQOL) and patient reported outcomes (PROs) in patients treated with PT for brain tumors. Adult patients with brain tumors treated with PT filled out the EORTC-QLQ-C30 and BN20 questionnaires up to three years following PT. Toxicity was scored using the CTCAE v4.03. QoL and PRO were correlated to clinical factors. Three-year overall survival, distant brain control and local control rates were 98%, 97% and 84%, respectively. No ≥G3 acute toxicity was observed. Late PT-related ≥G3 severe toxicity occurred in seven patients (5.7%). Lower global QoL scores after PT were significantly correlated to low Karnofsky performance status (KPS) before PT (*p* = 0.001), surgical complications before PT (*p* = 0.04) and progressive disease (*p* = 0.017). A low QLQ-30 summary score at one year follow-up was correlated to sex (*p* = 0.015), low KPS before PT (*p* < 0.001), and central nervous system symptoms before PT (*p* = 0.018). Reported QLQ-BN20 neurological symptoms were correlated to lower KPS at baseline (*p* < 0.001) and surgical complications before PT (*p* = 0.03). PT-related toxicity only influenced reported symptoms directly following PT, but not QoL. Although global QoL temporarily decreased after treatment, it improved again from one year onwards. Global QoL and reported symptoms over time were not correlated with the proton therapy and were more related to preexisting symptoms and progressive disease. This study assists in improving patient support in patients with brain tumors receiving PT.

## 1. Introduction

Radiotherapy (RT), with or without neurosurgery, is one of the main treatment modalities in the therapeutic armamentarium for patients with brain tumors, especially for those located in the skull base, infra-tentorial or orbital area. In these anatomical locations, complete resection is often not possible and patients are at substantial risk of developing additional complications following surgery. Additionally, the close proximity of multiple critical anatomical structures makes the treatment with RT of these tumors equally challenging. Therefore, a highly conformal RT technique is required to achieve steep dose fall-off at the edge of the target volume. Highly conformal therapy decreases the dose to surrounding structures and thus minimizes potential side-effects [1]. Proton therapy (PT) is a highly conformal RT technique, which plays an important role in the treatment of these patients [2]. Due to the Bragg peak, protons are able to achieve a low dose to surrounding structures by preventing dose distribution beyond the target volume. Because of this, PT is able to spare relatively more healthy brain tissue from radiation dose [3].

The benefits of extended survival after PT have to be balanced against the development of treatment-related toxicity. Especially for low-grade brain tumors, the long life expectancy of these patients makes it extremely important to minimize the occurrence of any radiation-induced toxicity [4]. Therefore, health-related quality of life (HRQOL) and patient-reported outcome (PRO) assessment play an increasingly important role as additional outcome measures in clinical trials for brain tumor patients [5,6,7]. HRQOL and PROs are able to assess subjective factors on patient’s functioning and well-being, in addition to objective outcome measures like survival and presence of toxicity. The aim of this study was to prospectively evaluate HRQOL and PROs in patients treated with PT for primary brain tumors and compare these data to oncological outcome, clinical parameters and physician-scored toxicity.

## 2. Materials and Methods

**Figure 1:**
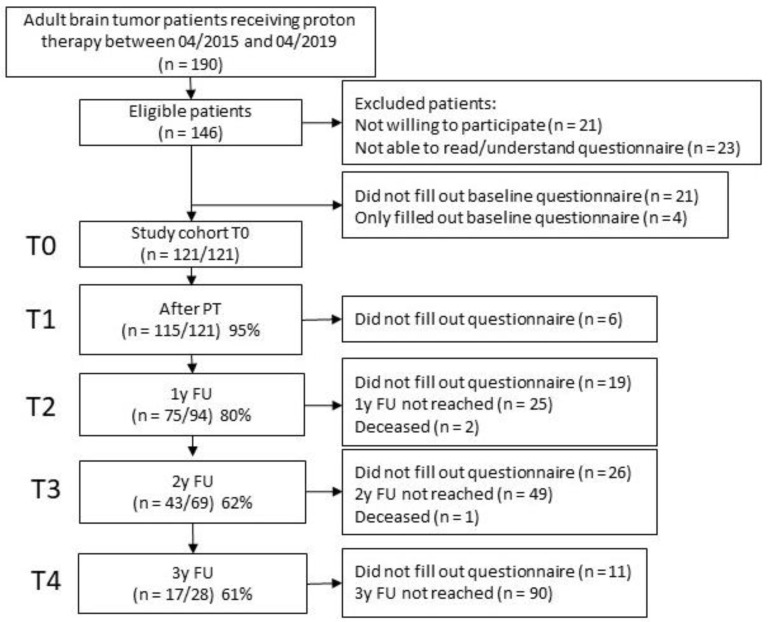
Flow diagram of patient inclusion and follow-up.

In total, 121/190 adult patients with primary brain or skull base tumors, treated with proton therapy (PT) at the Paul Scherrer Institute between April 2015 and April 2019, were included in this analysis. Reasons for patient exclusions are detailed in Figure 1. There was no significant difference in sex (*p* = 0.121), age (*p* = 0.943) or histology (*p* = 0.345) for included vs. excluded patients in this study. All participating patients filled out the EORTC-QLQ-C30 and BN20 questionnaires. This prospective study was approved by the ethics committee (EKNS-Nr. 2015-285) and written informed consent was obtained for all participants. Inclusion criteria were: Patients being ≥18 years of age, presence of a brain tumor receiving PT, no previous irradiation to the brain or head, no metastatic disease, no language barrier on understanding the patient brochure and good compliance to PT as well as to the study follow-up as assessed by the treating physician. There was a definitive indication for radiotherapy in all included patients, PT was determined individually for each patient in a multidisciplinary tumor board. The completed questionnaires were prospectively collected before (T0), directly following (≤7 days, T1), 1 year (T2), 2 years (T3) and 3 years (T4) following PT. All patients received follow-up visits after PT in 3 month intervals including clinical assessment as well as CT- or MRI-imaging. Additional ophthalmological, endocrinological, neurological and auditory examinations were performed as clinically needed. A paper version of the questionnaire was given to the patient at the start of PT and at the end of PT. After data entry, they were saved in an electronic database. During follow-up, questionnaires were sent to the participants per emails or by postal services if the patient was more comfortable with this delivery mode. A reminder was send to participants who did not complete/return the questionnaires within two weeks after initial notice.

### 2.1. EORTC QLQ-C30 and QLQ-BN20 Questionnaires

To evaluate HRQOL, the two validated EORTC questionnaires QLQ-C30 (version 3.0) and QLQ-BN20 were used [8,9,10]. The QLQ-C30 consists of 28 general questions grouped into 5 functional scales (physical, role, cognitive, emotional, social), 3 symptom scales (fatigue, pain, nausea/vomiting), global QoL and 6 items commonly reported in cancer patients (dyspnea, loss of appetite, insomnia, constipation, diarrhea and perceived financial impact of the disease). General symptoms and functional scales are scored as 1 = not at all, 2 = a little, 3 = quite a bit and 4 = very much and global QoL is scored from 1 = very poor to 7 = excellent. The QLQ-BN20 was developed and validated to specifically evaluate HRQOL of patients with brain tumors and consists of 20 questions analyzing 11 symptom scales (future uncertainty, visual disorder, motor dysfunction, communication deficit, headaches, seizures, drowsiness, alopecia, itchy skin, weakness of legs and bladder control). In the BN20, grading is defined as 1 = not at all, 2 = low, 3 = moderate and 4 = high. Following the EORTC QLQ-C30 and QLQ-BN20 scoring manuals, scores were calculated by averaging items within scales and transforming the average scores to a range between 0 and 100. High scores represent a higher level of functioning and HRQOL, or a lower level of symptoms. In the case of missing items within a scale, the scale score was calculated using the completed items present for the participant, but only if at least half of the respective items were completed. Additionally, the QLQ-30 summary score (SumSc) was calculated, as prescribed by Giesenger et al. [11] For the analysis of factors influencing the QLQ-BN20 score, all QLQ-BN20 neurological symptoms (visual disorder, communication deficit, headache, seizure, drowsiness, weak legs and bladder control) were combined into a summed score. Furthermore, alopecia and itchy skin were combined to compare skin toxicity. To determine the clinical relevance of changes in global QoL and SumSc scores, they were analyzed as introduced by Osoba et al. [12] Using this analysis, on a scale of 0 to 100, a change in the individual score at a certain time point compared to the baseline value of the same patient by 5–10 points indicates a small clinically relevant change; 10–15 points is a moderate clinically relevant change and >20 points is a highly clinically relevant change. This calculation system was used to identify either an improvement or a deterioration of symptoms per patient in our cohort.

### 2.2. Toxicity (CTCAE v4.03)

Acute (<3 months after PT) and late toxicity (≥3 months after PT) were prospectively scored using the Common Terminology Criteria for Adverse Events (CTCAE) version 4.03 [13]. Toxicity that was present at the time of first consultation was registered as tumor-related or prior surgery-related using medical records such as neurological-, ophthalmological-, and auditory examinations. Severe toxicity was defined as a CTCAE grade of ≥3.

### 2.3. Statistical Analysis

Statistical analysis was performed using SPSS v26.0 statistic software package (IBM Corp., Armonk, NY, USA). Kaplan–Meier survival curves with log-rank analysis for comparison of subgroups were used to evaluate overall survival (OS), progression free survival (PFS) and local control (LC). Friedman’s test was used to evaluate significant changes in reported scores over time. Differences between subgroups and correlations of the clinical parameters presented in Table 1 to global QoL, SumSc and BN20 neurological symptom scores were evaluated using the Mann–Whitney-U-Test and the Spearman’s rank correlation test. A *p*-value of less than 0.05 was considered statistically significant.

## 3. Results

### 3.1. Patient Characteristics

The response rate to the questionnaire was 80% after 1 year (75/94 patients), 62% after 2 years (42/69) and 61% (17/28) after 3 years. Most patients originated from Switzerland (67.0%) or Europe (29.6%). Only a minority of patients came from other continents, namely the Middle East (1.7%) or Australia (1.7%). The majority of patients (*n* = 109, 90.1%) had low grade tumors, mainly meningiomas WHO grade I-II, low grade chordomas or chondrosarcomas (Table 1). For 10 meningioma patients, no histological confirmation was available and the diagnosis was based on clinical presentation as well as MRI- and CT-imaging. Most tumors were located in the skull base (65.2%) and were partially resected (64.5%) at time of PT (Table 1). The timing of PT was directly after initial diagnosis in 79 patients (65%) and after a recurrence or progression of the tumor in 42 patients (35%). Patients underwent a median of 1 (range, 0–6) cranial surgeries before PT. A minority of patients (10.8%) received neoadjuvant, concurrent or adjuvant chemotherapy before, during and after PT. The median value of the planned target volume was 97.4 cc (range, 5.8–630.1 cc), and the median prescribed dose was 59.4 Gy (relative biological effectiveness (RBE)) (range 40.0–75.0 Gy (RBE). The median Karnofsky performance status (KPS) score was 100% (range, 60–100%) at t0 and remained unchanged with 100% (range, 60–100%) at T2.

### 3.2. Patient Survival and Treatment Response

The median follow-up was 24 months (range 1–45 months). Three patients died of tumor-related causes (2 astrocytoma cases, 1 MPNST). Overall survival was 99%, 98% and 98% after 1-, 2- and 3-years, respectively. During follow-up, 11 (9.1%) treatment failures were observed. In-field recurrences occurred in 8 patients (3 astrocytoma, 3 chordoma, 1 meningioma WHO grade II, 1 ependymoma patient), marginal failure in 1 patient (astrocytoma) and out-of-field local recurrences in 2 other patients (1 chordoma, 1 astrocytoma). The estimated 3-year LC was 93%, 90% and 84% after 1-, 2- and 3-years, respectively. Distant progression free survival was 97% after 1-, 2- and 3-years, respectively.

### 3.3. EORTC QLQ-C30 and QLQ-BN20 Scores

**Figure 2:**
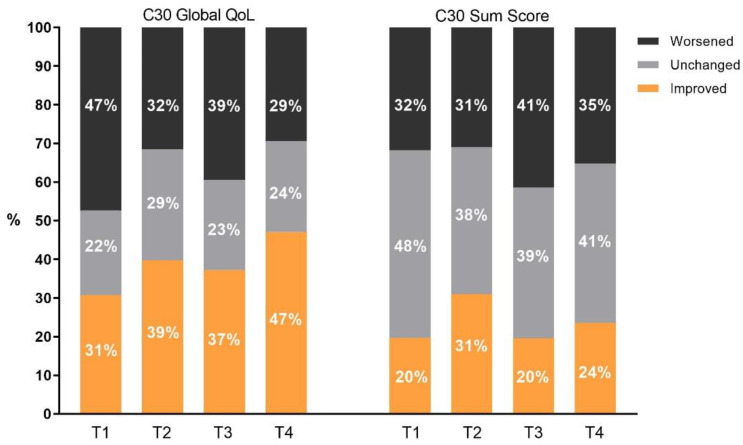
EORTC QLQ-C30 global QoL and C30 SumSc. The histogram shows the relative change in scores at T1 T1 (directly after PT), T2 (1-year follow-up), T3 (2-year follow-up) and T4 (3-year follow-up) compared to T0 (baseline directly before PT), as developed by Osoba et al. [12].

On an individual level, 47.4% of patients described a decrease in their global QoL directly following PT (T1), which returned to baseline levels 1 year later (T2). At T4, 57 (47.1%) patients noted an improvement in global QoL compared to baseline, whereas only 29.4% described a deterioration (Figure 2).

For the SumSc, 31.8% of patients reported a worsening directly following PT. However, after one year of follow-up (T2), 31% reported an improvement compared to baseline again. At 3 years after PT (T4), 35.3% reported a worsened, and 23.5% an improved, QLQ-30 SumSc compared to baseline (Figure 2). When comparing the whole cohort over time, global QoL and SumSc did not change significantly over the course of follow-up (Table S1). Furthermore, scores of all five QLQ-C30 functional scales did not change significantly during follow-up as well (Figure S1, Table S1). Of all general symptom scores of the QLQ C30, only fatigue and pain were regularly present directly before the start of PT; with 95 (79%) and 65 (54%) of patients reporting fatigue and pain, respectively. These symptoms remained present over time, and at 3-year follow-up (T4), 64.7% of patients still reported fatigue and 41.2% reported pain. However, both symptoms did not worsen significantly after the end of PT (Table S1). Furthermore, none of the symptoms reported in QLQ-BN20 changed significantly compared to baseline over the course of follow-up (Figure S2).

### 3.4. Toxicity

**Figure 3:**
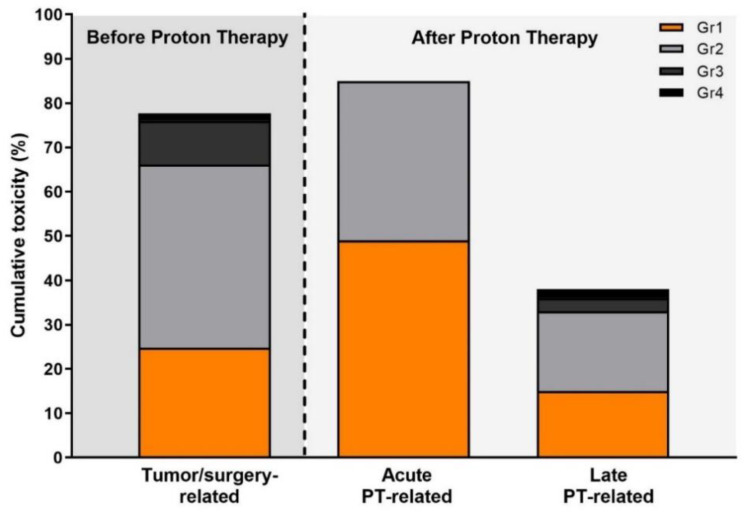
Percentage of the number of patients with observed tumor/surgery-related toxicity directly before proton therapy (PT), PT-related acute (≤3 months) and late (≥3 months) toxicity, graded by use of Common Terminology Criteria for Adverse Events (CTCAE) v4.03.

Toxicity data are presented in Figure 3. Before PT, 78% of patients presented with any form of neurological symptoms. These were caused primarily by the tumor in 66% or developed after surgery in 28% of patients and consisted of 16% severe toxicities (*n* = 9 patients reported optic nerve disorder, *n* = 8 auditory dysfunction, and *n* = 2 communication dysfunction). After PT, 83.5% of patients experienced mild (grade 1 or 2) acute toxicity. No severe toxicity was observed. Late toxicity caused by PT was present in 36% of patients, including 5.7% severe toxicities (grade 3:1 cataract, 4 auditory dysfunction, grade 4: 1 auditory dysfunction, 1 optical tract disorder) (Table S2).

### 3.5. Correlation of Clinical Parameters to Global QoL and PROs

Clinical parameters were compared to the global QoL, QLQ-C30 SumSc and QLQ-BN20 neurological symptom scores at 1-year follow-up (Table 2) and tested for correlation. Here, factors related to PT (radiation dose, acute toxicity, PTV volume) did not influence QoL and was only correlated to a lower SumSc and reported neurological symptoms directly following PT. Furthermore, there was no significant difference in QoL between patients that received gross tumor resection and PT compared to PT alone. Moreover, patients that were treated for a recurrent tumor did not have a significantly different OoL score compared to patients with a first tumor diagnosis (Table 2). Patients who reported a lower global QoL after 1 year had a significantly lower KPS at baseline, and more often surgical complications before PT or had progressive disease after PT. A low KPS before PT, the presence of CNS-symptoms before PT and female gender, adversely correlated with the QLQ-C30 SumSc after 1 year follow-up. QLQ-BN20 neurological symptoms after 1 year were worse for patients with a low KPS at baseline and surgical complications before PT.

## 4. Discussion

In this study, we were able to show excellent 2-year local tumor control rate of 90% and 2-year survival rate of 98%, with low rates of severe toxicity for brain tumor patients treated with PT. Importantly, global QoL scores were better than baseline values after acute toxicity had waned (from 1 year of follow-up onwards). The overall course of global QoL corresponds to other studies assessing global QoL after PT [14,15,16,17] and seems not to be the driving factor behind worsening neurocognitive functioning, which has been described after conventionally fractionated photon radiotherapy, e.g., in low-grade gliomas [10] or fractionated stereotactic treatment of skull-base meningioma [18,19]. Of all the factors contributing to these scores, PT did not significantly impair—nor did it improve—patient overall global QoL. When examining underlying factors, a correlation of lower global QoL scores to lower performance status and surgical complications before PT was observed. However, both were already impaired or present prior to PT (Table 2). Gender was found to influence the QLQ-C30 SumSc at 1 year of follow-up. A suggested reason for this could be a difference in coping and interpretation of symptoms, which has also been described by Elaldi et al. and Sehlen et al. [20,21] When evaluating individual symptom scores of the C30 and BN20, several remarkable observations were made, as they showed contradictory behavior to the global QoL scores. Visual disorder scores showed a trend to improve over time, although this was not significant. This is in line with previously published data for fractionated stereotactic radiotherapy in skull base meningioma [18]. Fatigue scores, on the other hand, were already lower than normal before PT and did not return to baseline even at 3 years FU. Interestingly, the physician-reported CTCAE fatigue score was comparable to scores reported by patients in the direct post therapeutic phase, but not in the later follow-up. Fatigue is a common observation in brain cancer patients and it seems to already have been present in a majority of patients before PT. The progressive fatigue during and following treatment is a known observation after radiotherapy and affected HRQoL in other brain cancer studies as well [16,22]. However, the fluctuation in fatigue that was observed over the 3 years of follow-up is more likely to be multifactorial [23]. Since endocrinological dysfunction is late-onset and rare within this time frame, it is not likely that this influenced fatigue in these patients [24]. It is thus suggested that fatigue is a subjective experience that is best assessed by self-reporting [23].

The functional scales of the C30 were more aligned with the SumSc and global QoL scores. Scores in these scales were slightly decreased directly after PT but generally went up to baseline one year after completion of PT. During follow-up, there were no significant decreases or increases. This finding is in accordance with studies examining Global QoL in adult and pediatric patients receiving PT for cranial tumors [6,14,15,16,17,25,26,27,28,29] and adult patients receiving stereotactic photon treatment for skull-base meningioma [18,19]. Studies investigating photon radiotherapy for low-grade gliomas, on the other hand, reported a decrease in neurocognitive functioning and consequently QoL over time [17,30]. The only evidence directly comparing photon and proton therapy, however, stems from a study on pediatric brain tumor patients showing a favorable effect on QoL after PT, further supporting the hypothesis of a positive influence of PT on QoL [17]. Interestingly, factors that negatively influenced global QoL in our cohort were most frequently tumor-related and were less often caused by PT. Most prominently, a low performance status before PT correlated significantly to a persistently worse global QoL after PT. Furthermore, PT-related (CTCAE) acute toxicity did not influence global QoL. On the other hand, patients who developed progressive disease after PT also reported a lower QoL. This was also found in several studies on glioma patients, where a decrease in reported QoL was thought to not be caused by the treatment, but by progressive disease outside the treated area [22,31].

To determine how PT can influence QoL in brain tumor patients, one has to look at its physical characteristics. PT irradiates less surrounding normal tissue compared to photon therapy, which partially reduces the acute and late side-effects of radiotherapy [31]. This is underlined by a study on pediatric brain tumor patients comparing these two entities, which found a favorable effect on HRQOL after PT [22]. Although the effects of PT on QOL in brain tumor patients are primarily investigated in pediatric patients [7,17,22,25,26,27,28], a number of studies on adults receiving PT have been performed as well [18,23,32]. Comparable to our results, these studies observed a decrease in QoL directly after PT, which improved again during follow-up. Another aspect where PT might be beneficial to PROs and QoL, is increased local control by the possibility of applying a higher radiation dose to the tumor. Patients in our study mostly presented with a skull base meningioma, chondrosarcoma or chordoma. Radiotherapy prolongs overall survival in these patients [33] and it was demonstrated that this is primarily influenced by local control, which is known to be better achieved with a higher radiation dose. In our cohort, a higher dose (≥ 70 Gy(RBE)) did not negatively influence global QOL, nor did it increase reported symptoms.

Challenges of our study lie in the evaluation of global Qol over time. Even though validation of the EORTC QLQ-C30 and BN20 questionnaires has been thoroughly performed, global QoL dynamics over time are often difficult to assess and interpret, as already noted by Osoba et al. [12] The observed increase in global QoL scores during FU can be the result of several factors. Although it is likely that PT positively influenced this improvement, another reason could be a response shift [34,35]. The subjective judgment of patients considering their QoL is known to change over time, not only due to the recovery of symptoms, but due to shifting personal expectations [36]. This is hinted at by a disparity between patients’ reported global QOL and their symptoms scored by QLQ-C30 SumScs. More patients reported improved global QoL than improvement in individual symptom scales. It appears that over time, ever more patients are able to cope with residual tumor- and treatment-related toxicities with less of an impact on daily living. This trend is further observed when surveying individual QLQ-C30 and BN20 symptom scores where, for example, stable neurological symptoms, but worsened future uncertainty over time, were reported. Another methodological challenge of QoL questionnaires is patient compliance during follow-up, as observed by other groups [37]. The current series managed a good compliance up to the 3-year follow-up [18,22]. Nevertheless, questionnaire response rates drop over long-term FU, putting studies at risk for bias due to the small number of responses obtained at later time points. As missing data can affect the interpretation of HRQoL assessed by questionnaire [38], we emphasized a structural distribution of the questionnaires to patients via post or email and sent them a reminder, while physician-assessed FU data continued to be collected. A further limitation of questionnaire-based assessment of patient-reported QoL is the trade-off between both breadth and depth questionnaires needed to be evaluated. On one hand, both the QLQ-C30 and BN20 generate a plethora of diverse and not necessarily cross-correlated data. We mitigated this risk by choosing Osoba scores and QLQ-C30 SumSC as well-validated methods of dimensionality reduction [11,12]. Both scores are also endorsed by EORTC, who developed the QLQ-C30 and BN20 questionnaire. On the other hand, each single item in the questionnaires—such as financial uncertainty—encompass broad aspects of patients’ lives, which can only capture a rough estimate of the patient’s subjective component of judging future development. Further focused investigation and differentiation into specific items will be necessary to avoid missing important aspects and to investigate the possible lack of correlation between global QoL scores and sums of symptom scales.

To elucidate and tackle this question, a prospective trial has been launched at our institution into financial “toxicity” burdened on patients by their disease and ensuing treatment (EKNS-Nr. 2019-01204). Moreover, further prospective trials are underway at other institutions; e.g., ProtoChoice-Hirn and Pro-CNS (www.clinicaltrials.gov NCT02824731 and NCT02797366 accessed on 15 May 2021) evaluating QoL with the QLQ-C30 and BN20 questionnaires. It will be of importance to the whole community to follow the results of these trials, especially following long-term survivors. Ultimately, prospective and comparative studies (e.g., between photon and proton radiotherapy) will be needed to build the evidence needed to guide and improve future treatment strategies.

## 5. Conclusions

In conclusion, PT resulted in an excellent 3-year survival and local control in brain tumor patients treated with PT. Although global QoL temporarily decreased after treatment, it improved again from one year onwards. Global QoL and reported symptoms over time were significantly related to preexisting symptoms and progressive disease and were not correlated to PT. This study could assist in improving patient support in patients with brain tumors receiving PT.

## Figures and Tables

**Table 1:** Patient and treatment characteristics.

Patient Characteristics:	N (%), Median (Range)
Age at time of proton therapy (years)	45.3 (18.8–77.7)
Sex (n):	
Female	84 (69)
Male	37 (31)
Histology (n):	
Meningioma	43 (35.5)
Chordoma	31 (25.6)
Chondrosarcoma	14 (11.6)
Astrocytoma	11 (9.1)
Oligodendroglioma	6 (5.0)
Pituitary gland adenoma	5 (4.1)
Ependymoma	3 (2.5)
Other *	8 (6.6)
WHO grade (n):	
I	76 (62.8)
II	33 (27.3)
III	2 (1.7)
Unknown	10 (8.3)
Tumor site (n):	
Skull base	79 (65.2)
Supratentorial	24 (19.8)
Infratentorial	3 (2.5)
Brain stem	4 (3.3)
Orbital	6 (5.0)
Sinus	5 (4.1)
Surgical status before proton therapy (n):	
Gross total resection	28 (23.1)
Partial resection	78 (64.5)
Biopsy only	5 (4.1)
No surgery	10 (8.3)
Nr of cerebral surgeries before proton therapy:	1 (0–6)
Nr of oncological surgeries before proton therapy:	1 (0–3)
Timing proton therapy (n):	
Initial diagnosis	79 (65)
Recurrence or progression	42 (35)
Time between diagnosis and proton therapy	9 (2–349)
Time between surgery and proton therapy	4 (1–203)
Chemotherapy (n):	
Neoadjuvant	3 (2.5)
Concurrent	3 (2.5)
Adjuvant	7 (5.8)
Planned target volume (cc) (median, range)	97.4 (5.8–630.1)
Proton therapy prescribed total dose (Gy):	59.4 (40–75)
Duration of radiotherapy (days)	47 (35–56)
KPS prior to proton therapy (%)	100 (60–100)
KPS at after finish of proton therapy (%)	100 (60–100)

**Table 2:** The association between EORTC QLQ-C30 global QoL, C30 SumSC and BN20 neurological symptom score and clinical parameters. PT = proton therapy, GTR = gross total resection. PTV = planning target volume. T0 = before PT, T1 = directly after PT and T2 = 1 year after PT. These time points are chosen as here the largest influence of PT is to be expected.

		T0			T1			T2		
Variables		Global QoL	C30 Sum Score	Neurological Symptoms	Global QoL	C30 Sum Score	Neurological Symptoms	Global QoL	C30 Sum Score	Neurological Symptoms
Sex (male, female)	*p* value	0.143	0.072	**0.04**	0.241	0.07	0.13	0.148	**0.015**	0.055
Age at start of PT	*p* value	0.827	0.349	0.231	0.154	0.25	0.291	0.343	0.922	0.968
Tumor WHO grade	*p* value	0.133	0.4	0.614	0.133	0.359	0.251	0.972	0.725	0.823
Timing of PT (first diagnosis, recurrence/progression)	*p* value	0.327	0.834	0.778	0.561	0.706	0.307	0.968	0.657	0.445
Resection status (GTR, R2/no surgery	*p* value	0.111	0.067	0.131	0.303	**0.033**	0.074	0.245	0.236	0.416
Karnofsky-Score at baseline	*p* value	**<0.001**	**<0.001**	**<0.001**	**<0.001**	**0.002**	**0.001**	**0.001**	**<0.001**	**<0.001**
CNS-symptoms pre-PT	*p* value	0.164	0.691	0.087	0.435	0.691	0.784	0.113	**0.018**	0.075
Surgical complications pre-PT	*p* value	**0.003**	0.062	0.091	0.118	0.571	0.09	**0.039**	0.086	**0.028**
Chemotherapy ((neo)adjuvant/concurrent)	*p* value	0.255	0.865	0.402	0.795	0.695	0.751	0.061	0.334	0.776
PTV volume (ml)	*p* value	0.786	0.744	0.722	0.345	0.199	0.834	0.947	0.667	0.774
Prescribed dose (Gy)	*p* value	0.5464	0.9	0.311	0.425	0.39	0.465	0.913	0.227	0.297
Acute PT-related toxicity	*p* value	0.919	0.508	0.721	0.079	**0.006**	**0.026**	0.724	0.349	0.29
Late PT-related toxicity	*p* value	0.891	0.335	0.645	0.666	0.522	0.65	0.472	0.338	0.236
Progressive disease in follow-up	*p* value	**0.012**	0.125	0.182	0.692	0.881	0.444	**0.017**	0.08	0.368

## Data Availability

The data presented in this study are available on request from the corresponding author. The data are not publicly available due PSI repository is currently work in progress.

## Supplementary Materials

The following are available online at https://www.mdpi.com/article/10.3390/cancers13194892/s1, Figure S1: EORTC QLQ-C30 function scores (mean ± SD) at baseline (T0), directly following PT (T1) and at 1 year (T2), 2 year (T3) and 3 year (T4) follow-up. Figure S2: EORTC BN20 function scores (mean ± SD) at baseline (T0), directly following PT (T1) and at 1 year (T2), 2 year (T3) and 3 year (T4) follow-up. Table S1: Friedman’s test to evaluate significant changes of reported scores from baseline to 3 year follow-up. A *p*-value of <0.05 shows a significant difference. Table S2: Overview of observed tumor/surgery-related toxicity directly before proton therapy (PT), PT-related acute (≤3 months) and late (≥3 months) toxicity, graded by use of Common Terminology Criteria for Adverse Events (CTCAE) v4.03. Numbers are represented as number of events (%).
