# Supplementary figures and images for: A Prospective Study on Health-Related Quality of Life and Patient-Reported Outcomes in Adult Brain Tumor Patients Treated with Pencil Beam Scanning Proton Therapy
Source: Cancers (Basel). 2021 Sep 29;13(19):4892. doi: 10.3390/cancers13194892 (PMC8507714; doi:10.3390/cancers13194892)

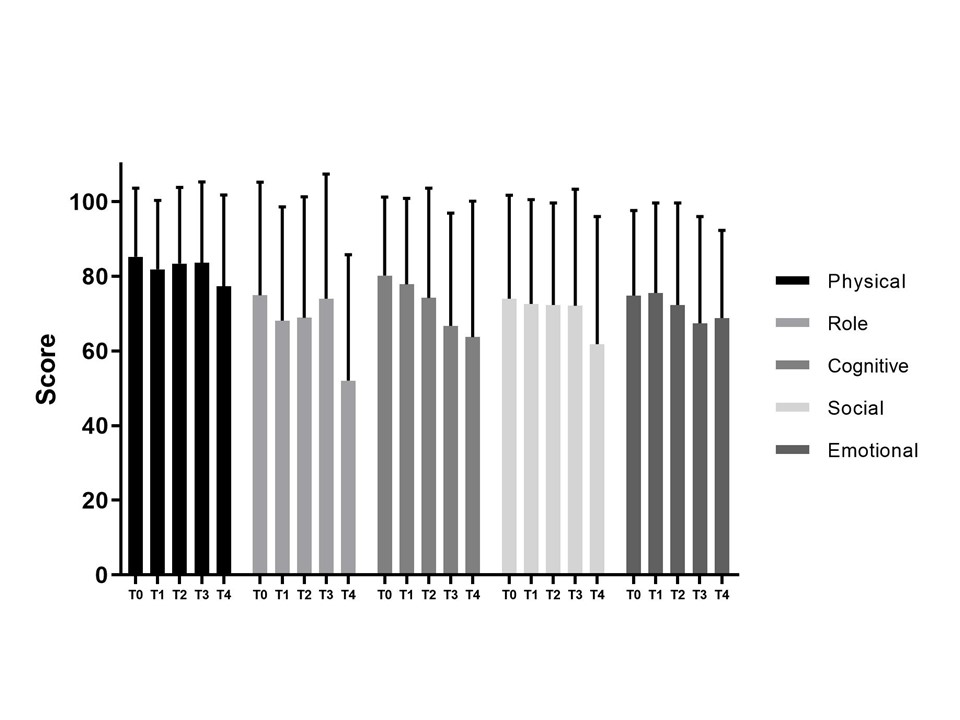

Supplement: Supplementary file 1 [file cancers-13-04892-s001.zip › Figure S1.jpg]

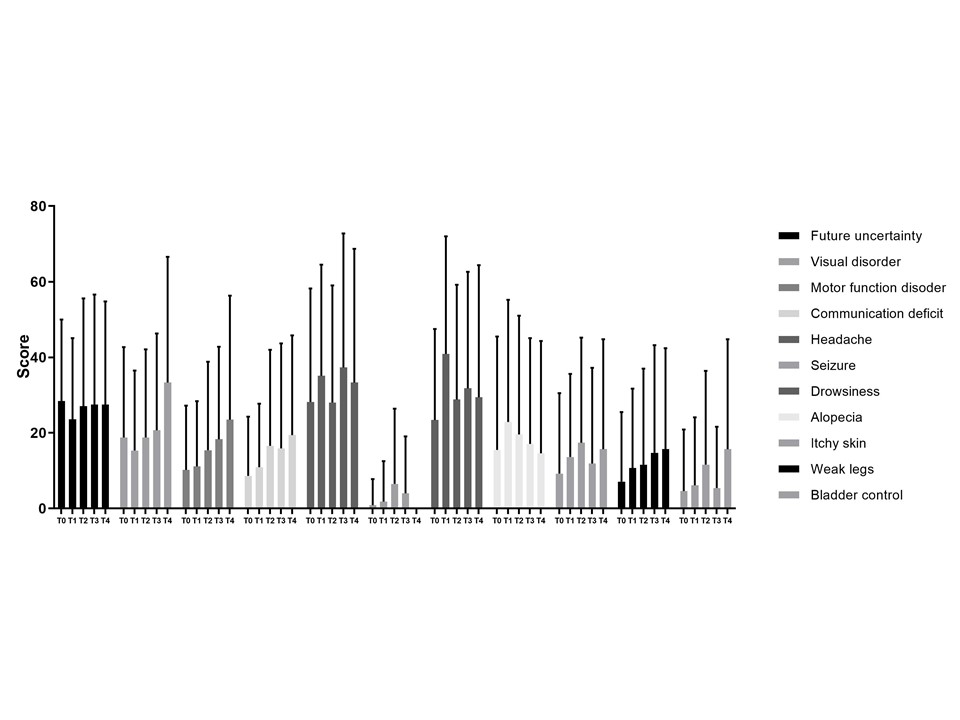

Supplement: Supplementary file 1 [file cancers-13-04892-s001.zip › Figure S2.jpg]
